# Multi-scale agent-based modeling on melanoma and its related angiogenesis analysis

**DOI:** 10.1186/1742-4682-10-41

**Published:** 2013-06-21

**Authors:** Jun Wang, Le Zhang, Chenyang Jing, Gang Ye, Hulin Wu, Hongyu Miao, Yukun Wu, Xiaobo Zhou

**Affiliations:** 1 College of Computer and Information Science, Southwest University, Chongqing 400715, China; 2 Department of Biostatistics and Computational Biology, Center for Biodefense Immune Modeling, University of Rochester, 601 Elmwood Avenue, Rochester, NY 14642, USA; 3 Department of Urology, Center of Nephrology, The Second affiliated Hospital of the Third Military Medical University, Chongqing 400037, China; 4 Center for Vaccine Development, University of Maryland School of Medicine, Baltimore, MD 21201, USA; 5 Department of Radiology, The Wake Forest University School of Medicine, Winston-Salem, NC 27157, USA

**Keywords:** Microenvironment, Drug synergism, Agent-based model, Multi-scale, Melanoma, Anti-angiogenesis

## Abstract

**Background:**

Recently, melanoma has become the most malignant and commonly occurring skin cancer. Melanoma is not only the major source (75%) of deaths related to skin cancer, but also it is hard to be treated by the conventional drugs. Recent research indicated that angiogenesis is an important factor for tumor initiation, expansion, and response to therapy. Thus, we proposed a novel multi-scale agent-based computational model that integrates the angiogenesis into tumor growth to study the response of melanoma cancer under combined drug treatment.

**Results:**

Our multi-scale agent-based model can simulate the melanoma tumor growth with angiogenesis under combined drug treatment. The significant synergistic effects between drug Dox and drug Sunitinib demonstrated the clinical potential to interrupt the communication between melanoma cells and its related vasculatures. Also, the sensitivity analysis of the model revealed that diffusivity related to the micro-vasculatures around tumor tissues closely correlated with the spread, oscillation and destruction of the tumor.

**Conclusions:**

Simulation results showed that the 3D model can represent key features of melanoma growth, angiogenesis, and its related micro-environment. The model can help cancer researchers understand the melanoma developmental mechanism. Drug synergism analysis suggested that interrupting the communications between melanoma cells and the related vasculatures can significantly increase the drug efficacy against tumor cells.

## Background

Melanoma is the most malignant skin tumor, causing the majority (75%) of deaths related to skin cancer [1]. Conventional diagnoses and treatments of Melanoma consist of surgical removal, chemotherapy, immunotherapy, and radiation therapy. The interferon can elongate the lifetime of the patient, but it can neither greatly increase the survival rate nor be a standard adjuvant treatment for melanoma [2].

Recently, several molecular drugs, such as doxorubicin and etoposide, were developed to treat melanoma cancer [3]. However, because of absorption, distribution, metabolism, or toxicity (ADME) problems, most molecular drugs did not work as well in vivo as in vitro. Many synergistic drug delivery methods have been developed to increase the drug effect in vivo, but it is difficult to quantitatively evaluate their performance. Thus, one of the aims of this study is to develop such indexes and tools that can estimate drug effects on melanoma cells.

It is known that angiogenesis [4-7] is a significant transforming phase in tumor growth. A drug’s distribution inside a tumor is highly heterogeneous due to the tumor vasculature’s tortuous, chaotic structure compared to fine, nearly parallel blood vessels in normal tissue. Drugs delivered to tissues will not only change the behavior of melanoma cells (secretion of cytokines, proliferation, differentiation, apoptosis, or migration) in the intracellular drug-triggered cell division process, but also inhibit the development of new capillary sprouts by preventing sprouts from receiving vascular endothelial growth factors (VEGF). In turn, inadequate glucose and oxygen transported from the blood vessel will drive even more melanoma cells towards apoptosis. Therefore it is of great necessity to take tumor-induced angiogenesis into consideration and simulate the irregular vasculature inside tumor in order to further study the drug distribution and drug therapeutic effects.

Many mathematical models [8-19] have been proposed to address the current challenges mentioned above. These models studied one or more phases of cancer progression, including tumor growth, angiogenesis, and drug treatment, with the purpose of better understanding the pathophysiology of cancer, mechanisms of drug resistance, and the optimization of treatment strategies. Although biologists have already obtained many experimental data sets at the molecular, cellular, micro-environmental and tissue levels, only a few scientists have integrated these data into a multi-scale platform to investigate the tumor progression with regard to its related angiogenesis and drug treatment. Studies on the anti-angiogenesis drug effects and the drug combination treatment responses are still rare.

Hence, this research presents a 3D multi-scale agent-based model to investigate the role of the tumor-angiogenesis interactions in melanoma tumor progression by extending our previously well-developed 2D agent-based tumor growth models [20-22]. The multi-scale system is comprised of intracellular, intercellular, and tissue levels to describe the melanoma growth with angiogenesis. As a rule based model, this study developed a set of rules to determine the melanoma cell’s phenotypic switch. These rules not only underline the migration of endothelial cells and the branching of vessel sprouts, but can also be more easily integrated into the agent based tumor growth model than previous Hybrid Discrete-Continuum (HDC) rules [23]. The model also can be employed as the test bed to predict the in vivo tumor responses to the combined drug pair: one for anti-angiogenesis and the other for the tumor.

In general, the multi-scale model can not only simulate melanoma tumor expansion with related angiogenesis, but also explore the best drug combination for tumor treatment and the dual role of angiogenesis (transporting both nutrient and drugs).

### Mathematical models

In order to describe tumor growth with angiogenesis and study melanoma’s response to given drug pairs, our model defines two types of agents: the melanoma cell and the endothelia cell. The melanoma cell and the endothelia cell agents represent the progression of tumor and vasculature, respectively. The aforementioned multi-scale model consists of three biological levels: the intracellular, intercellular, and tissue levels. The intracellular level describes the fundamental mechanism for cell’s phenotypic switch. The intercellular level bridges the tissue and intracellular scale as follows. (a) The vasculature delivers oxygen, cytokine, and glucose to the tumor microenvironment in the tissue level; (b) The melanoma cells uptake the glucose for metabolism as well as switch the phenotype under the stimulation of specific cytokine in the intercellular scale; (c) In turn, the inadequate glucose and oxygen will stimulate the tumor cell to secrete the VEGF in the intercellular level to induce angiogenesis. In the tissue level, blood vessel sprouts migrate and branch via tip endothelial cells’ migration in response to the diffused VEGF and drugs.

### Initialization

**Figure 1:**
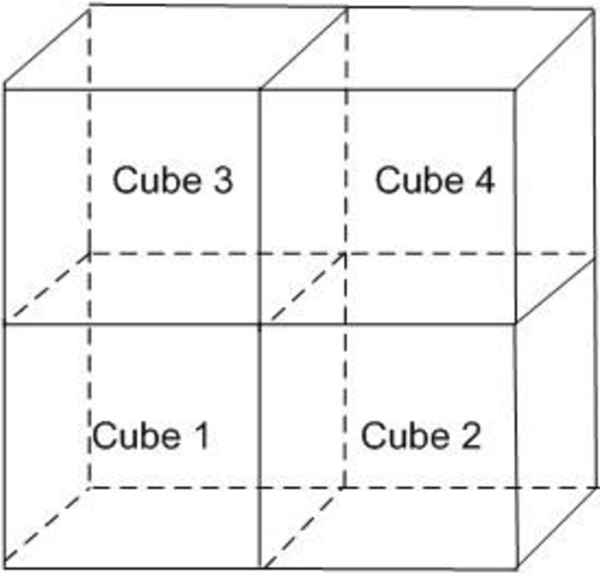
The 3D lattice represents the tumor extracellular matrix.

We use a 100×100×100 cube (Figure 1) with four sub-compartments to represent a slice of the virtual tumor extracellular matrix (ECM). The lattice size is 10 *μm*, which is approximately the same as the radius of the tumor cells. A hundred active melanoma cells are initialized in the center of the lattice like a sphere and the age of each tumor cell is randomly initialized between 0–24 hours. Sixteen tip endothelia cells are initialized on the surface of the 3D ECM as the main blood vessels. The VEGF and glucose are normally distributed in the cube 1 to 4 at the start, respectively.

### Intracellular level: the phenotypic switch of melanoma cells

**Figure 2:**
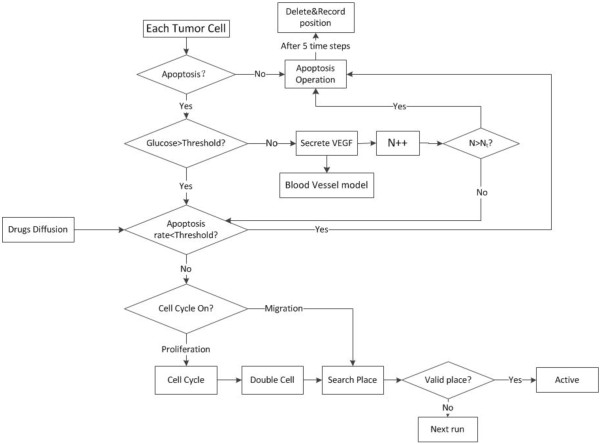
The flowchart for melanoma cell at intracellular scale.

At every simulation step (Δ*t* = 2 h*ours*), each melanoma cell determines its phenotype according to the following rules as shown in Figure 2.

### Apoptosis

If the concentration of the glucose is less than the cell’s survival threshold, the starving cancer cell will secrete VEGF to induce angiogenesis for nutrition deliver. If the starving cancer cell stays in an environment with inadequate nutrition for too long, it may go to apoptosis phenotype as Equation 1.

(1) $$\mathrm{Apo}p_{\mathrm{melrate}}\left(\mathrm{\Delta t}\right)=\left\{\begin{array}{c} 1-e^{-\lambda_{0}\mathrm{\Delta t}}\;\mathrm{without}\;\mathrm{drug}\\ 1-e^{-\left(\lambda_{0}+\lambda 1\right)\mathrm{\Delta t}}\;\mathrm{with}\;\mathrm{drug} \end{array}\right)$$

where *Δt* is the time interval and *λ*_*0*_ is the normal death rate of the melanoma cell. *λ*_*1*_ denotes the impact of the cytotoxic drug as Equation 2.

(2) $$\lambda_{1}=w_{1}A_{\mathrm{sDox}}/\left(1+w_{2}A_{\mathrm{sglu}\cos e}\right)$$

where *A*_*sDox*_*and A*_*sglucose*_ denote the average drug (Dox, which is a cytotoxic drug directly to melanoma cell) and glucose concentrations on the current site and its Von Neumann neighbors, respectively. *w*_*1*_, *w*_*2*_ are the regulatory factors.

Proliferation: Equation 3 describes the proliferation probability of the melanoma cell.

(3) $$p_{\mathrm{prol}}\left(\mathrm{\Delta t}\right)=1-e^{-\lambda_{2}}{}^{\left(\mathrm{\Delta t}\right)}$$

where *λ*_*2*_ is the normal proliferation rate of the melanoma cell equal to the reciprocal of average proliferation time of the cell.

Die function (Equation 4) is determining whether the cell enters the cell cycle or not.

(4) $$\left\{\begin{array}{c} C_{\mathrm{rand}\in \left[0,P_{\mathrm{prol}}\right)\;\mathrm{cell}\;\mathrm{cycle}\;\mathrm{ON}}\\ C_{\mathrm{rand}\in \left[P_{\mathrm{prol}},1\right)\;\mathrm{cell}\;\mathrm{cycle}\;\mathrm{OFF}} \end{array}\right)$$

with a die *C*_*rand*_ ∈ [0, 1), if the die *C*_*rand*_ falls into the interval [0, *p*_*prol*_), the cell enters the cell cycle and starts to proliferate.

### Migration

If the cell is neither in the cell cycle nor dividing, it will migrate. The detail of migration will be discussed in the next section.

### Quiescence

After the cell determines its phenotype, it will look for a free place that is of least resistance, most permission, and highest attraction [24] to divide or migrate. The cell will enter a reversible quiescent state in the absence of a free space.

### Intercellular level

Three major extracellular micro-environmental factors, such as glucose, VEGF, and drugs, are discussed in this model. A set of reaction–diffusion equations describes the diffusion, degrading, and uptake of these factors.

Glucose diffuses, degrades, and it is consumed by tumor cell as described in Equation 5

(5) $$\begin{array}{l} G_{\mathrm{ijk}}\left(t+1\right)=\{G_{\mathrm{ijk}}\left(t\right)\times \left(1-\lambda_{g}\right)+\lambda_{g}/6\times \sum \begin{array}{c} 6\\ l=1 \end{array}G\begin{array}{c} l\\ \mathrm{ijk} \end{array}\left(t\right)\\ \;+\chi_{\mathrm{endo}}\left(t,P_{\mathrm{ijk}}\right)*Pe_{g}-\chi_{\mathrm{tumor}}\left(t,P_{i}{}_{\mathrm{jk}}\right)*U_{g}\}*\left(1-D_{g}\right) \end{array}$$

where *G*_*ijk*_(*t* + 1) is the glucose concentration on the location *P*_*ijk*_ in the (t + 1) time step, *λ*_*g*_ is the diffusion constant of glucose. $G\begin{array}{c} l\\ \mathrm{ijk} \end{array}\left(t\right),l=1,2,\ldots ,6$ are the glucose concentrations of *P*_*ijk*_’s at its six immediate neighbors (Von Neumann neighbors) in the current time step. The time dependent characteristic functions *χ*_*endo*_ (*t*, *P*_*ijk*_) and *χ*_*tumor*_(*t*, *P*_*ijk*_) relate to the occurrences of an endothelia cell or a melanoma cell at *P*_*ijk*_. If a related cell is located at *P*_*ijk*_, the value of the function χ equals to 1; otherwise 0. *Pe*_*g*_ is the vessel permeability for glucose. *U*_*g*_ represents the glucose uptake rate of melanoma cell. *D*_*g*_ represents the natural decay rate of glucose.

The melanoma cell secretes VEGF to induce angiogenesis for the delivery of nutrients. VEGF diffuses in the surrounding tissue and is also consumed by the endothelial cells. This process is described by the following equation:

(6) $$\begin{array}{l} V_{\mathrm{ijk}}\left(t+1\right)=\{V_{\mathrm{ijk}}\left(t\right)\times \left(1-\lambda_{v}\right)+\lambda_{v}/6\times \sum \begin{array}{c} 6\\ l=1 \end{array}V\begin{array}{c} l\\ \mathrm{ijk} \end{array}\left(t\right)\\ \;+\chi_{\mathrm{tumor}}\left(t,P_{\mathrm{ijk}}\right)*Se_{v}-\chi_{\mathrm{endo}}\left(t,P_{\mathrm{ijk}}\right)*Pe_{v}\}*\left(1-D_{v}\right) \end{array}$$

Where *V*_*ijk*_(*t* + 1) is the VEGF concentration at the location *P*_*ijk*_ in the (t + 1) time step, *λ*_*v*_ is the diffusion constant of VEGF. $V\begin{array}{c} l\\ \mathrm{ijk} \end{array}\left(t\right),l=1,2,\ldots ,6$ are the VEGF concentrations of *P*_*ijk*_’s at its six immediate neighbors in the current time step. *Se*_*v*_ is the secretion rate for VEGF. *Pe*_*v*_ represents the vessel permeability rate of VEGF. *D*_*v*_ represents the natural decay rate of VEGF.

There are two drugs involved in our model. One is Doxorubicin (Dox) [25], which directly kills the tumor cells. The other is Sunitinib [26], which inhibits the growth of endothelial cells by preventing its receptor from receiving VEGF secreted from fast growing melanoma cells. After the drug is injected into the blood vessels, it is delivered through the vasculature and diffusing into the surrounding tissue. Finally, it is taken by the tumor cells and the endothelial cells. We model this process with the following equation:

(7) $$\begin{array}{l} DR_{\mathrm{ijk}}\left(t+1\right)=\left\{DR_{\mathrm{ijk}}\left(t\right)\times \left(1-\lambda_{d}\right)+\lambda_{d}/6\times \sum \begin{array}{c} 6\\ l=1 \end{array}\mathrm{DR}\begin{array}{c} l\\ \mathrm{ijk} \end{array}\left(t\right)+\chi_{\mathrm{endo}}\left(t,P_{\mathrm{ijk}}\right)*Pe_{d}\right\}\\ \;*\left(1-U_{d}\right) \end{array}$$

where *DR*_*ijk*_(*t* + 1) is the drug (Dox or Sunitinib ) concentration at the location *P*_*ijk*_ in the (t + 1) time step, *λ*_*d*_ was the diffusion constant of drug. $\mathrm{DR}\begin{array}{c} l\\ \mathrm{ijk} \end{array}\left(t\right),l=1,2,\ldots ,6$ are the drug concentrations of *P*_*ijk*_’s at six immediate neighbors in the current time step. *Pe*_*d*_ is the vessel permeability for drug. *U*_*d*_ represents the drug uptake rate.

As discussed before, once an agent (melanoma cell or endothelia cell) has determined its biomechanical phenotype, it will look for a free space to proliferate, migrate, or become quiescent. Each living melanoma cell chooses the “best” location to proliferate or migrate by the following rules:

1) Since the tumor cell always looks for a place with more nutrition to migrate to or to deliver its offspring to, we use the mean (*M*_*g*_) and the standard deviation (σ_g_) of the glucose concentrations on the place and its Moore neighbors [27] to locate candidate locations. Here, *G*(*P*^*l*^_*ijk*_) represents the glucose concentration of the *lth* Moore neighbors of the current site. If *G*(*P*^*l*^_*ijk*_) − *M*_*g*_ > *3σ*_*g*,_, we consider it as an abnormally high nutrition location for a tumor cell to migrate to or deliver its offspring to.

2) If *G*(*P*^*l*^_*ijk*_) − *M*_*g*_ ≤ *3σ*_*g*_, the model needs to evaluate all candidate locations nearby. All candidate locations were ranked through Equation 8.

(8) $$R_{l}=V_{l}*\left\{A_{\mathrm{mglucose}}^{l}/\left(1+w_{3}A_{\mathrm{mDox}}^{l}\right)\right\}$$

where *A*^*l*^_*mglucose*_ is the average glucose concentration of the *lth* candidate site and its Moore neighbors of this site. *A*^*l*^_*mDox*_ is the average Dox drug concentration of the *lth* candidate site and its Moore neighbors of this site. *w*_*3*_ is the regulator factor. The tumor cell always prefers a location that has a high nutrition concentration (*A*^*l*^_*mglucose*_), a low drug concentration (*A*^*l*^_*mDox*_), and few neighborhoods (*V*_*l*_). The preference of neighborhoods (*V*_*l*_) is denoted by Equation 9

(9) $$V_{l}=\left\{\begin{array}{c} 1/\mathrm{cellnum}\;\mathrm{cellnum}\in \left[7,26\right]\\ 1/6\;\mathrm{cellnum}\in \left[5,6\right]\\ 1/4\;\mathrm{cellnum}\in \left[3,4\right]\\ 1\;\mathrm{cellnum}\in \left[0,2\right] \end{array}\right)$$

Ranks of candidates were normalized as Equation 10.

(10) $${\tilde{R}}_{l}=\frac{R_{l}}{\sum_{l}R_{l}}$$

Normalized ranks formed the scale as Equation 11

(11) $$S=\left\{S_{l}:S_{l}=\left[\sum {}_{m=0}^{m=\left(l-1\right)}{\tilde{R}}_{m},\tilde{R}+\sum {}_{m=0}^{m=\left(l-1\right)}{\tilde{R}}_{m}\right)\right\}$$

*S* is an ordered set of *S*_*l*_. Each *S*_*l*_ is a region in the [0,1] and relates to the *lth* candidate site. The die casting generates a random *valued* ∈ [0, 1). If *d* falls in *S*_*l*_, the candidate location relates to the ${\tilde{R}}_{l}$ will be chosen as the next migration or proliferation stop.

3) If no space is available, the cell will become reversible quiescent.

### Tissue level

The starving melanoma cells secrete VEGF to induce angiogenesis and the induced vasculature transports nutrient for the tumor growth in the tissue level. Here, we employ the motion of the tip individual endothelial cell (“EC agent”) to represent vasculature progression.

**Figure 3:**
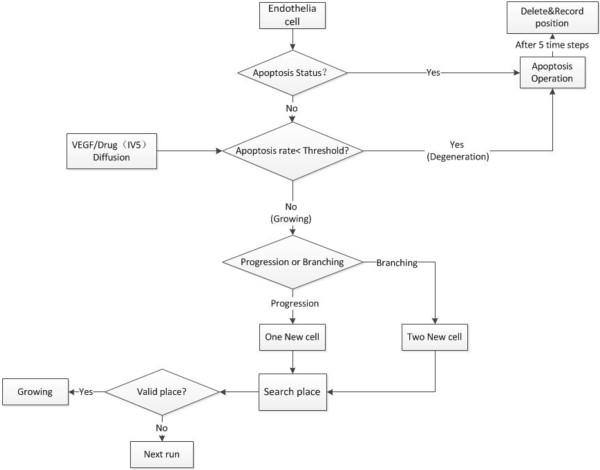
The flowchart for endothelia cell at tissue scale.

The algorithm for angiogenesis (Figure 3) is described as follows:

1) At each time step, each EC agent evaluates the VEGF concentration in its surrounding tissue. If there is no VEGF, the EC agent becomes quiescent.

2) Degeneration: The purpose of the drug Sunitinib is to inhibit tip endothelia cell’s ability to receive the VEGF signal as well as increase the apoptosis rate of tip endothelia cell. The threshold of a tip endothelial cell’s apoptosis rate (*Apop*_*endorate*_) is computed by Equation 12.

(12) $$\mathrm{Apo}p_{\mathrm{endorate}}\left(\mathrm{\Delta t}\right)=\left\{{}_{1-e^{-\left(\lambda_{s}+\lambda_{4}\right)\mathrm{\Delta t}}\mathrm{with}\;\mathrm{drug}}^{1-e^{-\lambda_{s}\mathrm{\Delta t}}\mathrm{without}\;\mathrm{drug}}\right)$$

where *λ*_*3*_ is the normal death rate of the endothelia cell and *λ*_*4*_ denotes the impact of the Sunitinib which is described by Equation 13.

(13) $$\lambda_{4}=w_{4}A_{\mathrm{sSuni}}/\left(1+w_{5}A_{s}{}_{\mathrm{VEGF}}\right)$$

where *A*_*sSuni*_*and A*_*sVEGF*_ denote the average Sunitinib drug and VEGF concentrations, respectively, on the current site and its Von Neumann neighbors. *w*_*4*_,*w*_*5*_ are the regulatory factors. At each time step, a uniformly random number is generated by the die function. If it is less than the apoptosis threshold, the endothelia cell becomes apoptotic and its parent cell is set as the tip endothelia cell.

Progression (migration): The living tip endothelia cell will proliferate or branch. The tip cell usually looks for the location with higher VEGF to branch to or proliferate to. The mean value *m*_*svegf*_ and the standard deviation *σ*_*svegf*_ of the VEGF concentration on the cell’s current site and its von Neumann neighbors are used to determine behavior of the tip endothelia cell *P*(*i*,*j*,*k*). Here, *V*(*P*^*l*^_*ijk*_) represents the VEGF concentration of the *l*th von Neumann neighbor of the current site. If *V*(*P*^*l*^_*ijk*_) − *m*_*svegf*_ > *3σ*_*svegf*_, we consider the VEGF is so strong that the blood vessel will directly grow toward this direction. If there are more than one candidate directions that meet the condition, the blood vessel will randomly select a direction to grow toward.

If the *V*(*P*^*l*^_*ijk*_) − *m*_*svegf*_ ≤ *3σ*_*g*_, the blood vessel tended to search valid spaces to branch.

3) Branching: For each EC agent, which tends to branch, their Moore neighbors are employed as candidate locations and ranked by Equation 14.

(14) $$R_{\mathrm{lendo}}=V_{l}*\left\{A_{\mathrm{mvegf}}^{l}/\left(1+w_{6}A_{\mathrm{mSuni}}^{l}\right)\right\}$$

where *A*^*l*^_*mvegf*_ and *A*^*l*^_*mSuni*_ are the average VEGF and average Sunitinib drug concentrations of the *lth* candidate site and its Moore neighbors of this site. *w*_*6*_ is the regulator factor. The endothelia cell always moves to a location with high VEGF, low drug concentration, and low crowdedness (*V*_*l*_) as described in Equation 9. All *R*_*lendo*_ were normalized by Equation 10. All normalized ranks were incorporated to form a scale *S* as specified by Equation 10. The die casting generates two random value *sd*_1_ ∈ [0, 1), *d*_2_ ∈ [0, 1). If *d*_*1*_, *d*_*2*_ fall in *S*_*l1*_, *S*_*l2*_, the candidate location which relate to the ${\tilde{R}}_{l1}$, ${\tilde{R}}_{l2}$ will be chosen as the branching sites. If both *d*_*1*_, *d*_*2*_ fall in the same region, the algorithm will repeat the die casting process.

4) If no space is available, cells would remain in a reversible quiescent state and try again in the next round.

**Figure 4:**
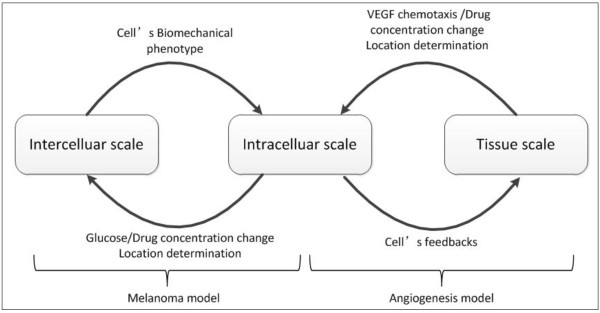
**Multi**-**scale Agent base model of melanoma and its related angiogenesis.**

This multi-scale agent based melanoma cancer model with angiogenesis is summarized as follows (Figure 4). At the intracellular level, it employs exponential functions (Equations, 1–4) to describe the phenotypic (migration, proliferation, or apoptosis) switch of the cancer and endothelia cells. At the intercellular level, a set of reaction–diffusion equations (Equations, 5–7) is employed to describe the spatial concentration changes of glucose, VEGF, and drugs. Cancer cells compete for the best location in the 3D extracellular matrix in order to migrate or proliferate depending on the gradient of glucose, drugs, and cell density (Equations, 8–11). At the tissue level, the spatial concentration distributions of VEGF and drug concentrations play an important role to impact the tip endothelial cells’ migration and sprout branching (Equations, 12–14). In turn, the dynamic vasculature at the tissue level remodels the tumor microenvironment by changing the important factors (spatial concentration distributions of glucose and drugs) in the intracellular level. And the behaviors of melanoma cells (secretion of cytokines, proliferation, migration, or apoptosis) are greatly influenced by these changes at the intracellular level. The parameters of the model are listed in Table 1.

**Table 1:** The parameters of the ABM

**Symbol**	**Variable**	**Initial value**	**Reference**
*λ*_*0*_	Normal death rate of melanoma cell	0	[28]
*w*_*1*_	Regulatory factor of Equation 1	0.01	Estimated
*w*_*2*_	Regulatory factor of Equation 1	1	Estimated
*λ*_*2*_	Initial proliferation rate of melanoma cell	32 h (16.9 h-47.3 h)	[28]
*λ*_*g*_	Diffusion constant of glucose	6.7 (5.2-7.2) × 10^-7^ cm^2^ /s	[22,29]
*Pe*_*g*_	Permeability for glucose	3 × 10^-5^ (0.2-7)cm/s	[22,30]
*U*_*g*_	Glucose uptake rate of melanoma cell	0.28 mmol/h	[22]
*D*_*g*_	Natural decay rate of glucose	0.2 (0.1-0.4)	Estimated
*λ*_*v*_	Diffusion constant of VEGF	2.9 (1–9.4) × 10^-7^ cm^2^/ s	[23,31]
*Se*_*v*_	Secretion rate for VEGF	0.6 (0.6-1) nM/h	[32]
*Pe*_*v*_	Permeability for VEGF	0.1 × 10^-4^ cm/s	[23,31]
*D*_*v*_	Natural decay rate of VEGF	0.2 (0.1-0.4)	Estimated
*λ*_*d*_	Diffusion constant of drug	5.18 (1–10) × 10^-7^ cm^2^ /s	[33,34]
*Pe*_*d*_	Permeability for drug	3 (0.2-7) × 10^-5^ cm/s	[33,34]
*U*_*d*_	Drug uptake rate	0.2 (0.1-0.4)	Estimated
*w*_*3*_	Regulator factor of Equation. 8	0.01	Estimated
*λ*_*3*_	Normal death rate of endothelia cell	0	[31]
*w*_*4*_	Regulatory factor of Equation 13	0.01	Estimated
*w*_*5*_	Regulatory factor of Equation 13	1	Estimated
*W*_*6*_	Regulatory factor of Equation 14	0.01	Estimated

## Results

We have implemented the above model in the VC++ programming environment. It includes a 3D melanoma-angiogenesis interaction model and its related drug combination treatment. We can employ this tool to predict the responses of melanoma and its related angiogenesis under drug combination treatment.

### Volumetric growth dynamics

We measured the tumor system’s (total) volume by counting the number of the lattice sites occupied by a tumor cell regardless of its phenotype, hence lumping together both proliferative and migratory expansion.

**Figure 5:**
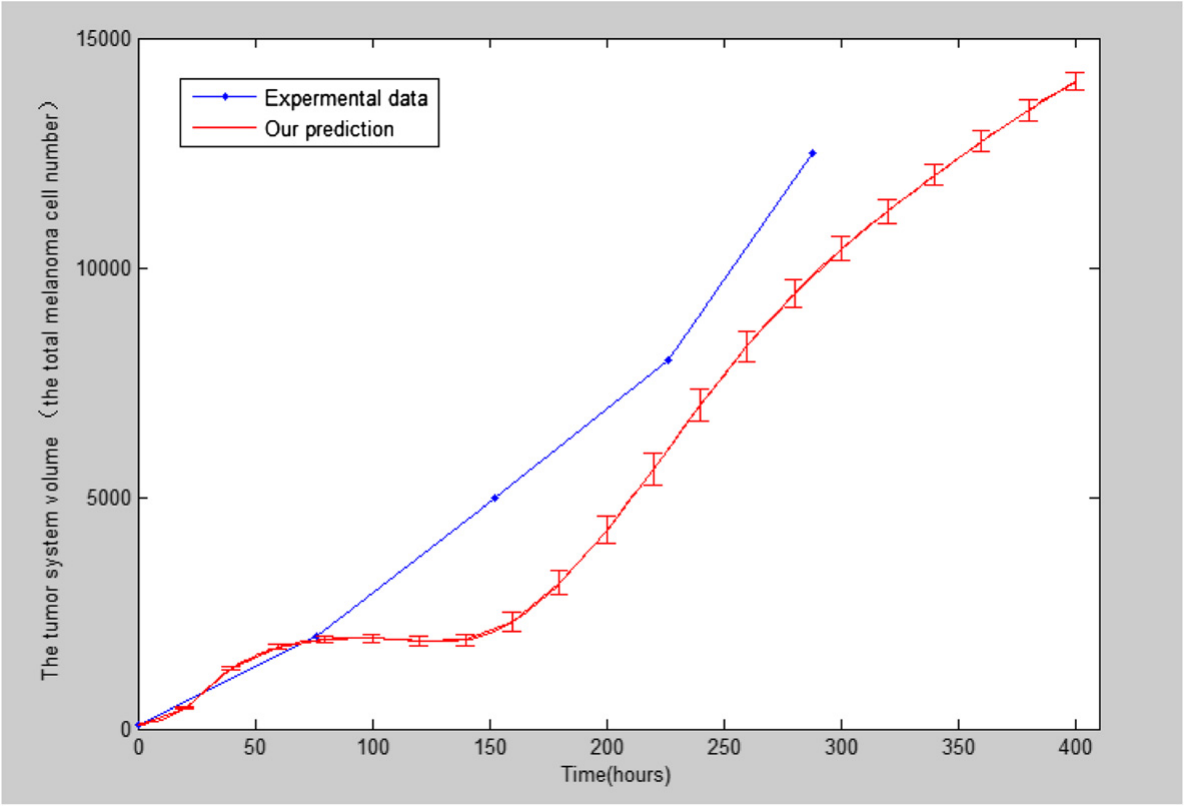
**The tumor system volume (y-axis) over time (x-axis) derived from simulations (red line, for t = 0-400 hours) and from*in vitro*experimental observations (blue line, for t = 0-300 hours).**

Figure 5 shows the increase in melanoma system volume over time for 20 simulation runs. The volume increase is not smooth. The volume quickly increased and slowed down during time interval between 50 h and 150 h. After that, the volume kept increasing rapidly. The simulated data is presented by a red line of Figure 5. As reported by Khodadoust et al. [35], there are two experimental data sets related to human melanoma, which were estimated at the indicated times by manual counting. The mean value of these two experimental data sets is the blue line of Figure 5, which has similar growth trend as the simulated data (red line).

### Tissue scale behavior

**Figure 6:**
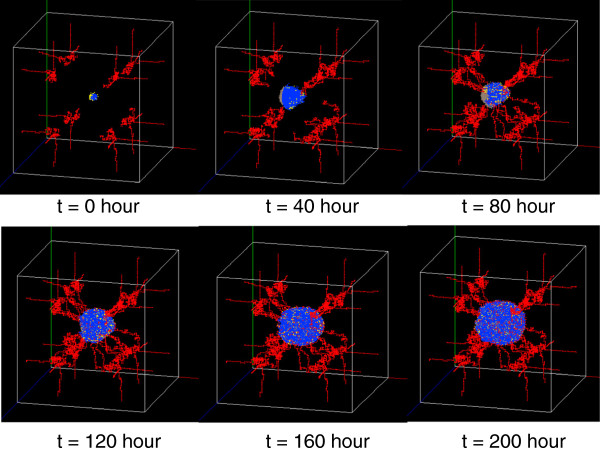
**3D snapshots of the tumor system at time step t=0, 40, 80, 120, 160, 200 hours.**

Figure 6 shows the three-dimensional snapshots of the tumor at time points 0, 40, 80, 120, 160 and 200. Note that different colors denote various tumor cell states: active (both proliferative and migratory) (yellow), quiescent (blue), dead (grey). The endothelial cells are red. Each time step is 2 hours. Figure 6 shows that melanoma cells tend to proliferate or migrate to the locations near the vessels, where the glucose concentration level is the highest. In the beginning, the tumor was comprised of active cells and some quiescent cells. The blood vessels were far away from the tumor. At around 40 hours, some dead cells appeared in the center of tumor and the blood vessels moved to the tumor. From 80 to 200 hours, not only did the number of both dead and active cells keep increasing, but also did the number of blood vessels that were approaching the tumor. Furthermore, more and more quiescent cells near the blood vessels switch the phenotype back to migration or proliferation. Finally, the blood vessels become a tree bunching microvasculature and were much denser near the tumor.

### Phenotypic behavior

**Figure 7:**
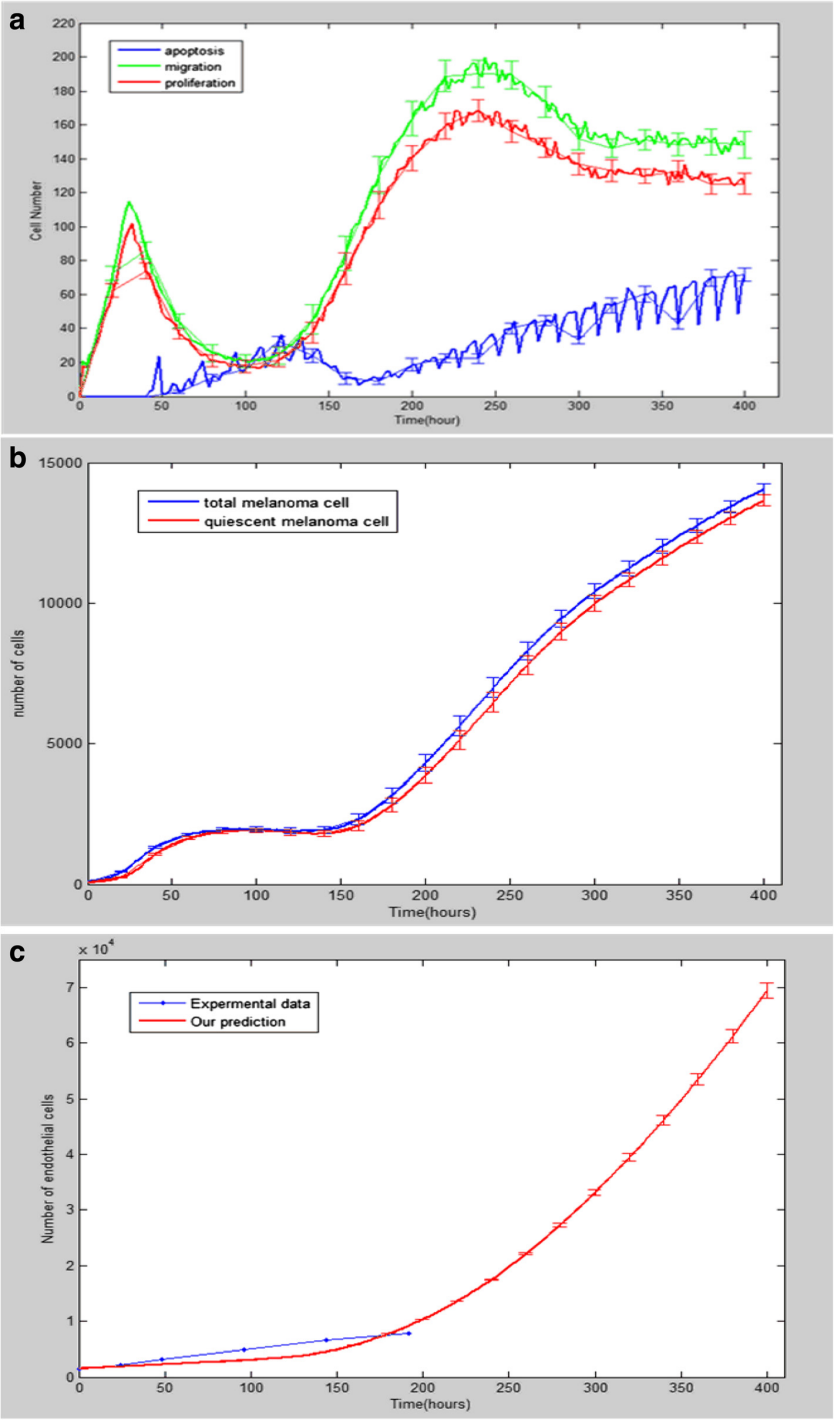
**Simulations of vascular melanoma growth without drug treatment.** (**a**) Different types of melanoma cell numbers from 0 hour to 400 hours. (**b**) The quiescent melanoma cell number compared with the total melanoma cell number from 0 hour to 400 hours. (**c**) The comparison between our model simulation results (red line, for t = 0-400 hours) and the *in vitro* experimental observations (blue line, for t = 0-200 hours) of endothelia cell number growth.

**Figure 8:**
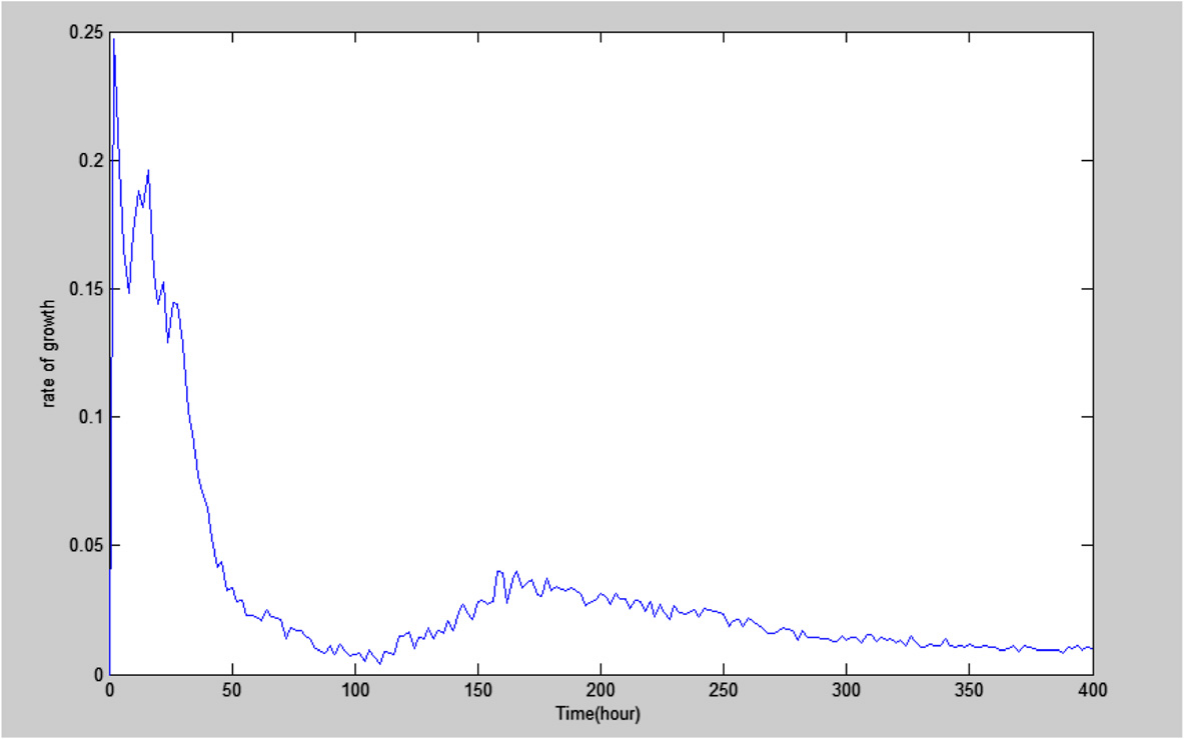
**Proliferation rate of melanoma cells derived from simulations for t** **=** **0**-**400 hours.**

Figure 7 shows the mean value and standard deviation of the numbers of different types of melanoma cells and endothelia cells with respect to time with 20 simulation runs. Also, Figure 8 presents the proliferation rate of tumor cells. Figure 7a shows that the number of active cells increased rapidly from 0 to 50 hours and decreased in [50 h, 150 h]. After that, the number of active cells increased monotonically with time until 250 hours. The increasing trend of active cells became mild after 250 hours. The number of apoptotic cells increased abruptly at around 50 hours and kept increasing until the tumor microvasculature developed around 150 hours. There was a significant decrease of apoptotic cells at around 150 hours. After that, the number of apoptotic cells kept to a relatively flat, monotonically increasing rate until 400 hours. Figure 7b shows that the number of quiescent melanoma cells kept increasing from 0 to 400 hours with a similar curve as the total melanoma cells. Figure 7c shows the comparison between the dynamics of the simulated and experimental number of the endothelial cells under angiogenesis condition [36]. The 200 hours’ experimental data (blue line) shows similar growth trend as the simulated data (red line).

### Combined drug effects on melanoma treatment

**Figure 9:**
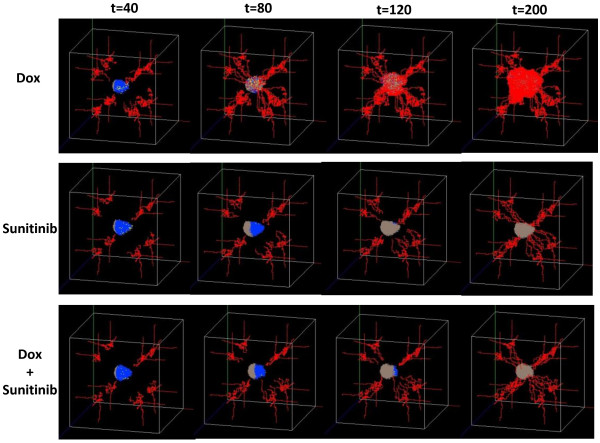
**3D snapshots of the tumor system on the drug treatments.** Single Dox/Sunitinib and the combination of Dox and Sunitinib treatments at time step t = 40, 80, 120, 200 hours.

We employed the drug Dox to directly increase the apoptosis rate of melanoma as well as used Sunitinib to treat melanoma by interrupting the VEGF signal communications between the melanoma and its related angiogenesis. Figure 9 shows 3D snapshots of the tumor system at time steps 40, 80, 120, 200 hours under Dox, Sunitinib, and the combined drugs (Dox and Sunitinib) treatments, respectively. The different colors represent cell types in the same manner as Figure 6. Figure 9 shows that Sunitinib is more effective than Dox at decreasing the tumor expansion, and the effect of combined drugs treatment is the best.

### Cell dynamics change under drug treatment

**Figure 10:**
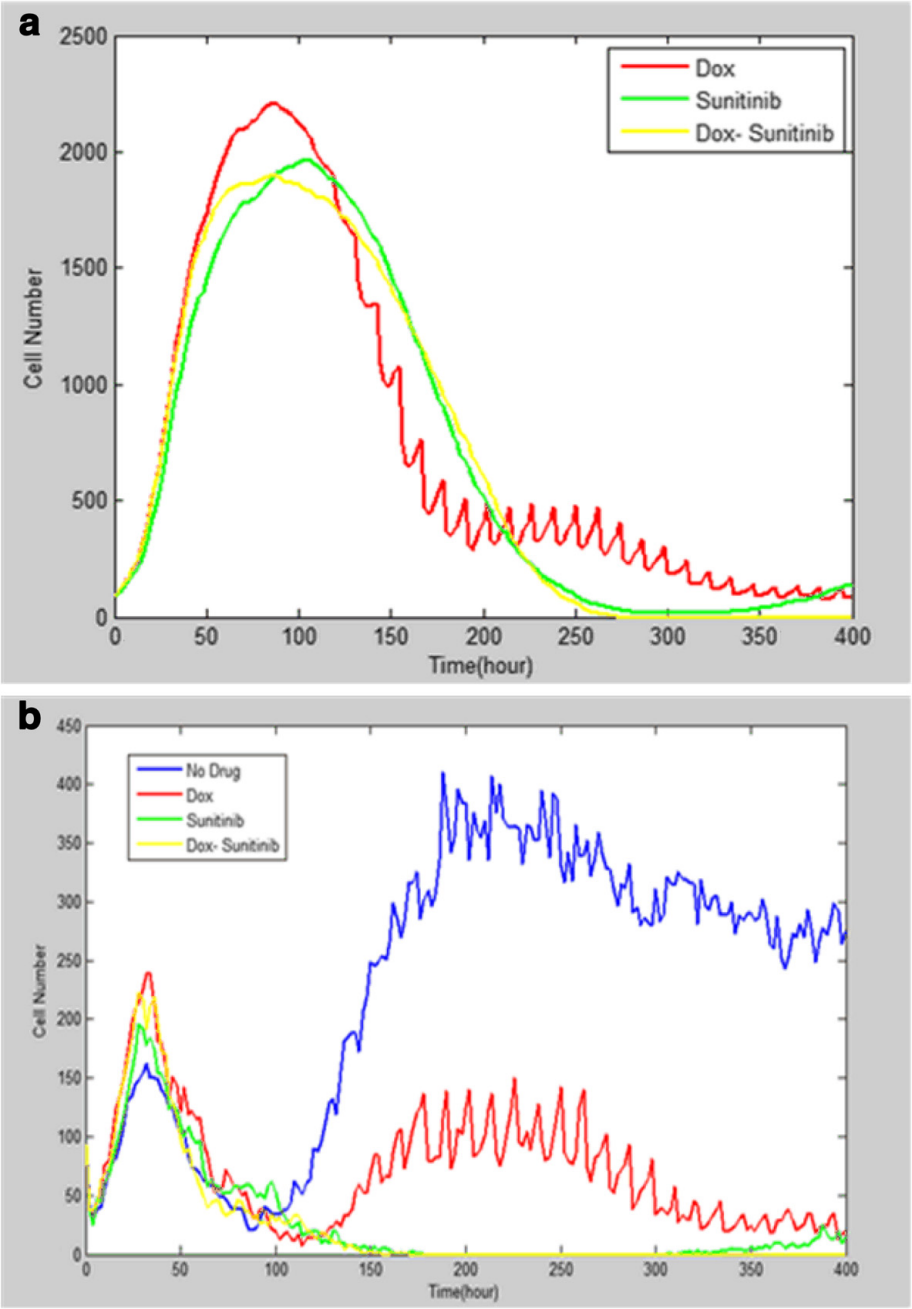
**The effects of drug treatments on tumor growth.** (**a**)The growing trends of total melanoma cells under different drug treatments (**b**) The growing trends of active tumor cells with or without drug treatments.

Figure 10 shows the average cell dynamics under different drug treatments in 20 simulations. Figure 10a shows a sharp drop of total melanoma cells at around 100 hours regardless of what the drug treatment was. Figure 10b shows that the number of the active melanoma cells started to drop around t = 50 hours which is earlier than the total tumor cells. In general, Figure 10 demonstrates that single Dox/Sunitinib therapy cannot kill all the tumor cells like the combined drugs therapy during the simulation.

### Drug effect test

**Figure 11:**
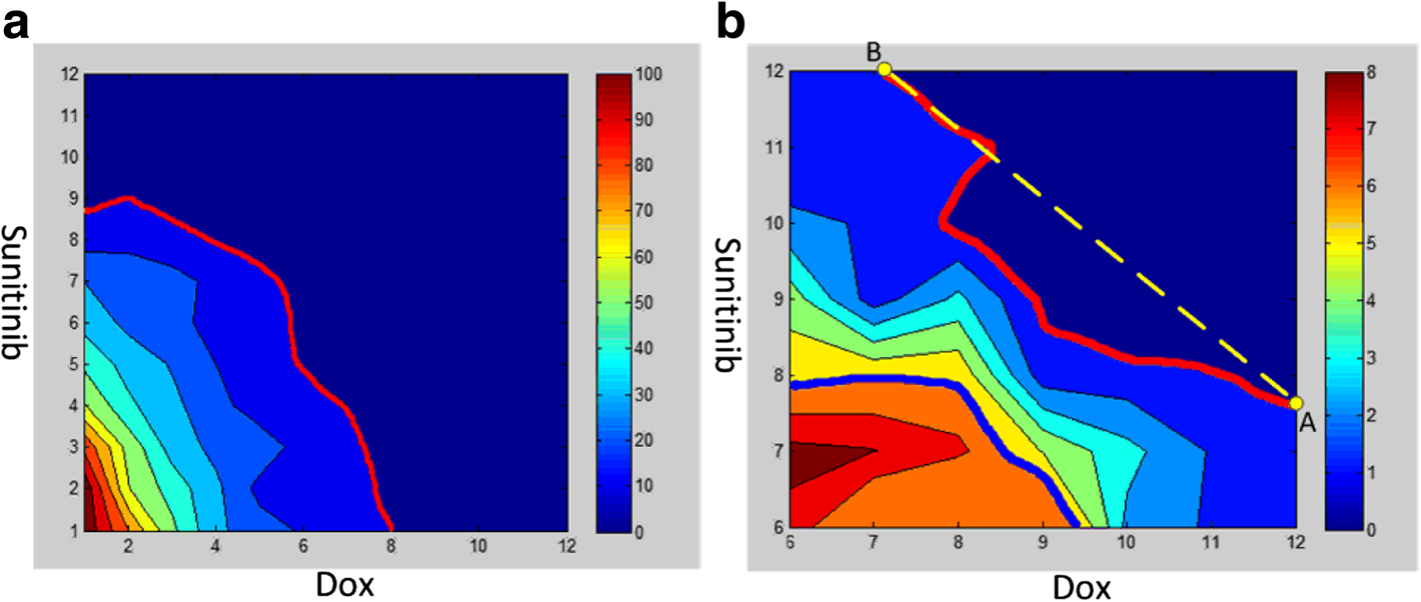
**Synergy Effects of Dox and Sunitinib on total melanoma size.** (**a**) the whole drug effects of Dox and Sunitinib combination (**b**) the details of the drug effects of Dox and Sunitinib combination in dose 6 to 12.

There were 12 doses for each drug, with 1 being the control and having 12 levels ranging from 0.1× to 10× in geometric sequence relative to the original dose. Then, we explored the drug efficacies for various combinations of the two drugs. We used the total melanoma cell death rate as the indicator of drug efficacy. Dramatic synergistic effects of the Dox and Sunitinib were observed if the total elimination of melanoma cells (E100 at time point 400 hr) was used as the criterion for Loewe combination index [37-39]. Figure 11a shows the whole effect of drug combinations, and the red line indicates that the number of the total tumor cells is lower than the initial number 100. The metric level of the color bar in Figure 11 is 1:10. That means that the value 10 indicates that the number of cancer cells is 100. Since Figure 11a cannot accurately describe the details of drug effect when the number of the total tumor cells is less than 100, Figure 11b is used to describe the dramatic synergistic effects of the Dox and Sunitinib when both drug doses are greater than 6. In Figure 11b, the blue line indicates the E50 isobole (the total number of the melanoma cells is lower than 50), and the red line marks the E100 isobole (the total elimination of melanoma cells). The yellow dashed line AB in Figure 11b represents the Loewe additivity criteria. The two end points A, B represent the concentrations of single Dox or Sunitinib with respect to the total elimination of melanoma cells. According to the Loewe additivity concept [37-39], if the E100 isobole is lower than the Loewe additivity line AB, the combination of the drugs has a synergistic effect; if the E100 isobole is higher than the Loewe additivity line AB, the combination of the drugs has an antagonistic effect; otherwise we say that the combination of the drugs has an additive effect.

### Parameter sensitivity analysis

To evaluate the impact of the parameter values on the behavior of the melanoma-angiogenesis system, we analyzed the sensitivity of our model to the following parameters: *λ*_*2*_, *λ*_*g*_, *Pe*_*g*_, *D*_*g*_, *λ*_*v*_, *Se*_*v*_, *D*_*v*_, *λ*_*d*_, *Pe*_*d*_, *U*_*d*_. We varied each parameter individually over the ranges shown in Table 1, while fixing other parameters constant at their base values. The ranges of the parameters are from the previous literatures listed in Table 1. Limited by the relatively high computing cost of the ABM, we did 10 simulations for each set of parameters. To assess the influence of the parameters, we calculated the Spearman rank-order correlation [40] of each parameter versus the number of total melanoma cells, the number of active melanoma cells, and the number of endothelia cells. Table 2 shows the Spearman rank-order correlations *ρ*, and *p*-*values* for each parameter. Also, it explores three parameters closely related to the endothelial cells number, total, and active melanoma cells number: the permeability for glucose (*Pe*_*g*_), the diffusion constant of drug (*λ*_*d*_), and the drug uptake rate, *U*_*d*_. The secretion rate for VEGF (*Se*_*v*_) closely correlates with endothelial cells number and active melanoma cells number, and it turns out that VEGF plays important role in the tumor growth and angiogenesis. However, the natural decay rate of glucose (*D*_*g*_) is only closely related to active melanoma.

**Table 2:** **Spearman rank**-**order correlations and *p***-**values between model parameters and simulation outcomes**

	**Total melanoma cells**		**Active melanoma cells**		**Endothelia cells**	
**Parameter**	**Spear *ρ***	***p*-value**	**Spear *ρ***	***p-*value**	**Spear*ρ***	***p*-value**
*λ*_*2*_	−0.1000	0.7698	0.9091	1.0559 E-4	0.1091	0.7495
*λ*_*g*_	0.1818	0.5926	0.1455	0.6696	−0.3545	0.2847
*Pe*_*g*_	0.9818	8.4031 E-8	0.9818	8.4031 E-8	0.9000	1.5997 E-4
*D*_*g*_	−0.7273	0.0112	−0.9613	2.4489 E-6	−0.1636	0.6307
*λ*_*v*_	0.3455	0.2981	−0.0182	0.9577	0.3818	0.2466
*Se*_*v*_	0.5550	0.0770	0.6320	0.005	0.6640	0.0260
*D*_*v*_	−0.1273	0.7092	0.31434	0.3465	0.1182	0.7293
*λ*_*d*_	0.9818	8.4031 E-8	0.9431	1.3526 E-5	0.9727	5.1422 E-7
*Pe*_*d*_	−0.0909	0.7904	−0.0365	0.9153	0.6182	0.0426
*U*_*d*_	0.9909	3.8406 E-9	0.9677	1.0925 E-6	1	0

### Discussion and conclusions

This study proposed a 3D multi-scale agent-based cancer model by integrating a novel angiogenesis module into a tumor growth module. The major aims of the research are investigating the relationship between the melanoma-induced angiogenesis, melanoma development, as well as exploring the optimum synergistic drug combinations to treat melanoma cancer.

The inadequate nutrition in the microenvironment will decrease the tumor proliferation rate (Figure 8) and make the melanoma cell undergo apoptosis (Figure 5 and Figure 7). Starving melanoma cells will release VEGF to induce the angiogenesis for nutrition. In turn, the angiogenesis will promote the tumor growth (Figure 6). This study developed such a platform that can estimate optimum drug dose combinations for tumor treatment. Figure 9 and Figure 10 intuitively showed that the anti-angiogenesis drug (Sunitinib) has much better effect than the tumor-specific cytotoxic drug (Dox), which directly kills the melanoma cells. Moreover, the synergistic use of both Sunitinib and Dox can significantly decrease melanoma progression and inhibit tumor-induced angiogenesis, since the drug combination therapy can not only kill the cancer cells by increasing the apoptosis rate, but also inhibit the cancer cells’ ability to obtain the enough nutrients by interrupting communication between the cancer cells and the vasculature. The Drug effect test based on the Loewe drug combination analysis also strongly suggested that combination of cytotoxic drug (Dox) and the angiogenesis inhibitor (Sunitinib) are of high clinical potentials. Classical Loewe combination analyses often use isobole at effect level 50 (E50) as standard index, which is convenient to monitor in animal models and clinical chemotherapy evaluations. Due to the advantages of simulations, the E100 isobole was employed to evaluate the performance of drug combinations in killing all melanoma cells in a given treatment time. Simulations of the combination effects indicated the drug combinations could successfully kill almost all melanoma cells in the given treatment time (the red lines in Figure 11a and b). When it was evaluated by the E100 isobole against the melanoma cells, the simulations also suggested strong synergistic effects (the red line in Figure 11b is lower than the Loewe additivity criteria dashed line). As shown in Figure 11b, without the aid of Sunitinib, Dox alone cannot extinguish all melanoma cells and thus enabling the disease to relapse soon. Taken together, angiogenesis is highly targetable during melanoma treatment, and inhibitors of the interactions between cancer initiating cells and their related angiogenesis are promising co-drugs for traditional cytotoxic agents. The sensitivity analysis not only explored the high correlation between simulation outcomes and blood vessel delivery rates of glucose and drugs, but also demonstrated that interrupting communication between the tumor and its related angiogenesis can significantly amplify the effect of the treatment.

This is the first time a 3D multi-scale agent-based cancer model was employed to describe the communication between the melanoma and the vasculature around the tumor and investigate how to employ anti-angiogenesis drugs to cure melanoma by breaking this communication. This study also indicated that angiogenesis plays a very important role in the transportation of nutrients for the tumor growth. Drug synergism analysis indicated that inhibiting communications between melanoma cells and their related vasculature could increase the efficacy of the treatment, decrease the tumor progression, and finally reduce the cancer cell survival rate. In the distant future, we are going to develop a predictable cancer model by considering more realistic biological and physical data and features, such as blood flow, the influence of focal adhesion kinases, complicated signaling pathway, and the oxygen pressure [41].

## Abbreviations

ABM: Agent based model; ECM: Extracellular matrix; VEGF: Vascular endothelial growth factor; Dox: Doxorubicin.

## Competing interests

The authors declare that they have no competing interests.

## Authors’ contributions

JW and LZ carried out the MABM studies, participated in the model design and drafted the manuscript. CYJ implemented the computing algorithms. YG, YKW, HW, HM and XZ did algorithm development and improved the manuscript. All authors read and approved the final manuscript.
